# A Pilot Study of Neurobiological Mechanisms of Stress and Cardiovascular Risk

**DOI:** 10.18103/mra.v11i4.3787

**Published:** 2023-04-25

**Authors:** J. Douglas Bremner, Marina Piccinelli, Ernest V. Garcia, Valeria M. Moncayo, Lisa Elon, Jonathon A. Nye, C. David Cooke, Brianna P. Washington, Rebeca Alvarado Ortega, Shivang R. Desai, Alexis K. Okoh, Brian Cheung, Britt O. Soyebo, Lucy H. Shallenberger, Paolo Raggi, Amit J. Shah, Obada Daaboul, Mohamed Nour Jajeh, Carrie Ziegler, Emily G. Driggers, Nancy Murrah, Carlo N. De Cecco, Marly van Assen, Robert T. Krafty, Arshed A. Quyyumi, Viola Vaccarino

**Affiliations:** 1 Department of Psychiatry & Behavioral Sciences, Emory University School of Medicine, Atlanta, GA; 2 Department Radiology and Imaging Sciences, Emory University School of Medicine, Atlanta, GA; 3 Department Medicine (Cardiology), Emory University School of Medicine, Atlanta, GA; 4 Department Biomedical Informatics, Emory University School of Medicine, Atlanta, GA; 5 Department of Epidemiology, Emory University, Atlanta, GA, USA; 6 Biostatistics and Bioinformatics, Rollins School of Public Health, Emory University, Atlanta, GA, USA; 7 Atlanta VA Medical Center, Decatur, GA, USA; 8 Mazankowski Alberta Heart Institute and the Department of Medicine, University of Alberta, Edmonton, Alberta, Canada.

**Keywords:** stress, PTSD, cardiovascular disease, depressive disorders

## Abstract

**Objective:**

Coronary heart disease is a leading cause of death and disability. Although psychological stress has been identified as an important potential contributor, mechanisms by which stress increases risk of heart disease and mortality are not fully understood. The purpose of this study was to assess mechanisms by which stress acts through the brain and heart to confer increased CHD risk.

**Methods:**

Coronary Heart Disease patients (N=10) underwent cardiac imaging with [Tc-99m] sestamibi single photon emission tomography at rest and during a public speaking mental stress task. Patients returned for a second day and underwent positron emission tomography imaging of the brain, heart, bone marrow, aorta (indicating inflammation) and subcutaneous adipose tissue, after injection of [^18^F]2-fluoro-2-deoxyglucose for assessment of glucose uptake followed mental stress. Patients with (N=4) and without (N=6) mental stress-induced myocardial ischemia were compared for glucose uptake in brain, heart, adipose tissue and aorta with mental stress.

**Results:**

Patients with mental stress-induced ischemia showed a pattern of increased uptake in the heart, medial prefrontal cortex, and adipose tissue with stress. In the heart disease group as a whole, activity increase with stress in the medial prefrontal brain and amygdala correlated with stress-induced increases in spleen (r=0.69, p=0.038; and r=0.69, p=0.04 respectfully). Stress-induced frontal lobe increased uptake correlated with stress-induced aorta uptake (r=0.71, p=0.016). Activity in insula and medial prefrontal cortex was correlated with post-stress activity in bone marrow and adipose tissue. Activity in other brain areas not implicated in stress did not show similar correlations. Increases in medial prefrontal activity with stress correlated with increased cardiac glucose uptake with stress, suggestive of myocardial ischemia (r=0.85, p=0.004).

**Conclusions:**

These findings suggest a link between brain response to stress in key areas mediating emotion and peripheral organs involved in inflammation and hematopoietic activity, as well as myocardial ischemia, in Coronary Heart Disease patients.

## Introduction

Coronary heart disease (CHD) is associated with considerable morbidity and mortality.^1^ Stress^2-6^ and associated psychiatric disorders,^7-17^ including major depression^17-27^ and post-traumatic stress disorder (PTSD)^9,17,28-33^ are associated with an increased risk for CHD and adverse cardiac outcomes, but the reasons for that are unknown.^34-36^ Efforts to improve cardiac outcomes through interventions focused on treatment of stress-related disorders, like major depression, including psychotherapies and antidepressant medications, have met with limited success.^37-41^ A better understanding of mechanisms by which stress is associated with CHD risk is needed to create better interventions.^15,35,42,43^

Mental stress paradigms can be modelled in the laboratory to investigate mechanisms mediating the effects of stress on adverse cardiac outcomes.^44^ Stress, depression, and associated emotional factors such as anger can activate autonomic, inflammatory, and vascular responses, precipitating cardiac events.^45-53^ Mental stress can be measured in daily life as well as modelled in the laboratory using mental stress tasks like public speaking and mental arithmetic, and these studies showed that acute psychological stress can induce myocardial ischemia in some patients with CHD.^17,46,54-67^ CHD is linked to both depression^7-17,68^ and PTSD,^9,17,28-33^ and stress may mediate its effects either directly,^2-6^ through these psychiatric disorders^34,44,49,55,69-71^ or via a common genetic link.^72-75^ Mental Stress Ischemia (MSI) can occur in CHD patients without exercise-induced myocardial ischemia MSI is not necessarily associated with atherosclerotic CHD ^56,59,60,62-64,76-81^, is twice as common in women under 50 than similar aged men,^62^ and is associated with increased long-term risk for adverse cardiac events compared to conventional exercise-induced myocardial ischemia.^44,64,70,82-86^

The brain plays a central role in mediating the effects of stress on CHD.^34-36,42,43,87-90^ For stress to mediate an increased risk for CAD, the information related to the stressful event (visual, olfactory, auditory) has to come in through the senses and be processed by primary sensory cortices before it is relayed to a brain network mediating the stress response.^42,88,89,91-96^ Brain areas with outputs to the periphery, including the medial prefrontal cortex, amygdala (via the lateral nucleus of the hypothalamus), and insula, activate peripheral sympathetic, neurohormonal, cardiovascular, and inflammatory responses to stress, which facilitate survival.^87^ Understanding how these interconnected systems respond to stress could be useful in developing interventions for patients with CHD. [^18^F]2-fluoro-2-deoxyglucose (FDG) is a radiolabeled form of glucose which can be imaged with positron emission tomography (PET). FDG is taken up in the brain similar to glucose, which is the primary energy source of the brain. It is also taken up in other areas similar to glucose, including areas of ischemia in the heart, and active areas of inflammation and bone marrow activity. The purpose of this study was to use PET FDG to study brain, heart, aorta (inflammation) and bone marrow responses to mental stress in patients with CHD.

## Methods

### Study Sample

Patients between the ages of 30 and 79 with known coronary heart disease (CHD) from the Mental Stress Ischemia Prognosis Study (MIPS) and the Mental Stress and Myocardial Ischemia after MI-2 (MIMS-2) studies who participated in a followup study in 2020-2021 were included. MIPS and MIMS-2 patients were originally recruited from Emory University Hospital, Grady Memorial Hospital and the Atlanta VA Medical Center from September 2010 to September 2020.^44,97^ CHD was defined based on a previous cardiac catheterization showing atherosclerosis, history of prior myocardial infarction, a history of percutaneous coronary intervention or coronary artery bypass grafting at least one year prior to the study, or a positive nuclear stress test. Patients were excluded if they had had a recent acute coronary syndrome, or decompensated congestive heart failure within 1 week of the enrollment visit, pregnancy based on pregnancy testing, systolic blood pressure greater than 180 mm Hg or diastolic blood pressure greater than 110 mm Hg on the day of the test, a history based on the Structured Clinical Interview for the Diagnostic and Statistical Manual IV (SCID) of a severe mental disorder including schizophrenia, psychotic depression, bipolar disorder, or alcohol or substance dependence in the past year, history of loss of consciousness of more than one minute, history of neurological disorder, such as dementia, stroke, or Parkinson’s Disease, or contraindications to regadenoson administration. Beta-adrenergic antagonists were held for 24 hours and calcium channel blockers and nitrates for at least 12 hours prior to the stress test. Patients for whom withholding medications was considered unsafe were excluded. All patients provided written informed consent, and the study was approved by the Emory University Investigational Review Board (IRB).

### Psychometric Assessment

All patients were assessed with a number of psychometric instruments, including the Beck Depression Inventory (BDI), a reliable and validated self-report measure of depressive symptoms.^98^ Information about medications and other clinical data were obtained through questionnaires and medical chart review. The Subjective Units of Distress Scale (SUDS) was used to assess stress before and after the stress procedures. Psychiatric diagnosis was assessed using the Structured Interview for the Diagnostic and Statistical Manual-IV (SCID).^99^

### Mental Stress Testing

Participants initially underwent cardiac imaging of the heart at rest and during a public speaking task. On a separate day participants returned for imaging of the brain during two mental stress tasks (mental arithmetic and public speaking) using methods previously described.^100^

### Cardiac Imaging at Rest and with Mental Stress

Participants underwent cardiac single photon emission computed tomography (SPECT) imaging of the heart for assessment of myocardial perfusion at rest and with mental stress. For the rest image they received 10-14 mCi of [Tc-99m] sestamibi intravenously. Thirty to 40 minutes later resting SPECT images of the heart were obtained at rest. Participants then underwent a public speaking task (nursing home scenario) following which they were injected with 10-14 mCi [Tc-99m]sestamibi at the time of peak stress followed in 30-40 minutes by SPECT imaging of the heart with mental stress. We have found these methods of measuring mental stress-induced myocardial ischemia to be highly reproducible ^101^.

### PET Whole Body Imaging with Mental Stress

**Figure 1.**
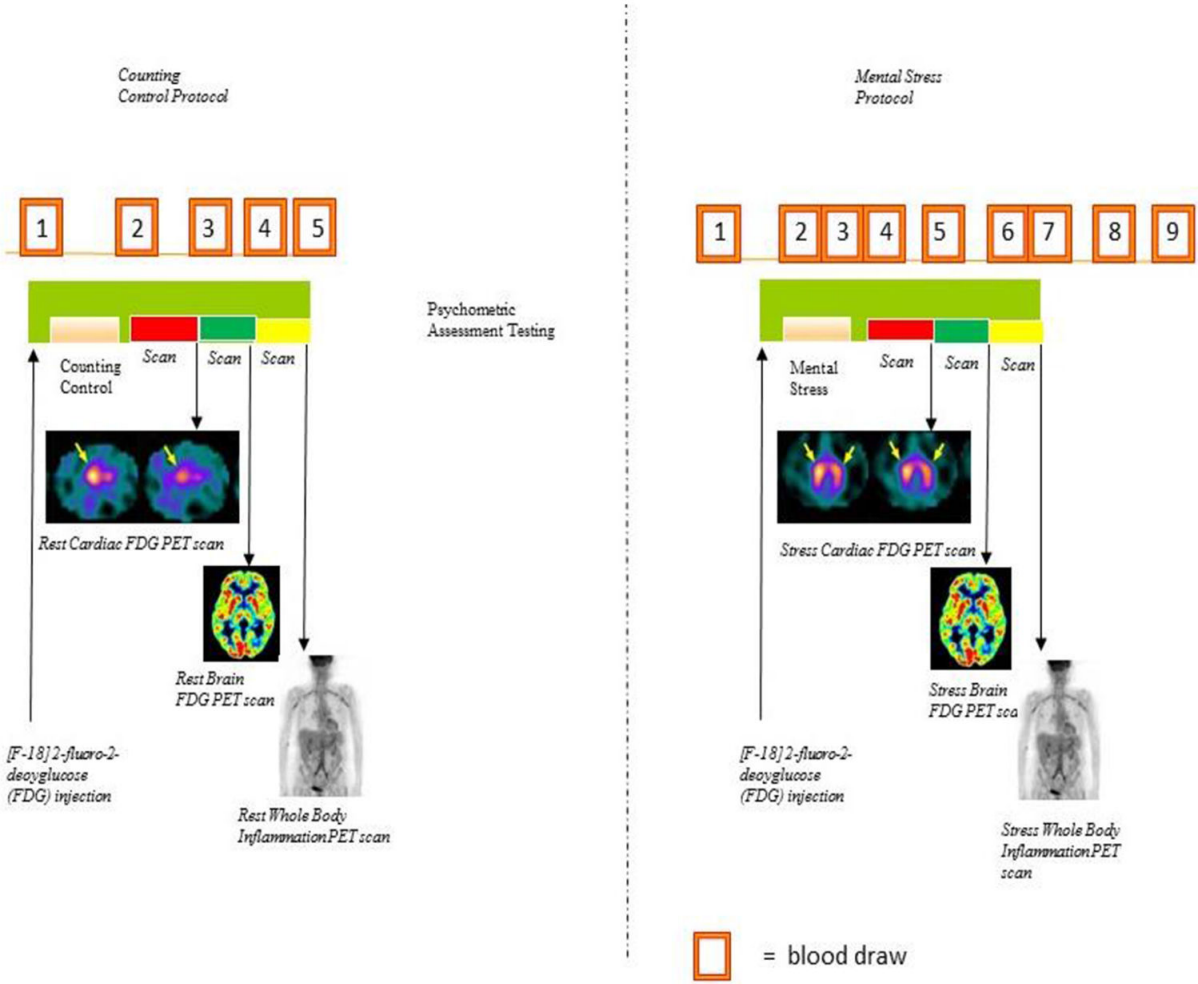
Cardiac, Brain and Inflammation Imaging Study Protocol. Patients receive FDG intravenously at baseline followed by counting control and then cardiac, brain and inflammation PET-CT imaging after a 30 minute FDG uptake period. The protocol is repeated with mental stress tasks. FDG cardiac scans show uptake in yellow where myocardium is ischemic, greater with stress. FDG brain functional scans show red/yellow activity in cortex. Increased FDG uptake is seen in the thoracic arteries indicating inflammation and FDG activity tracks bone marrow activity as well. The CT is used for volumetric assessment of subcutaneous and visceral adiposity.

Patients underwent positron emission tomography (PET) imaging of the brain, heart and whole body in conjunction with control and mental stress tasks (Figure 1). Based on the prior cardiac study 10 participants were selected including those with (N=4) and without (N=6) mental stress-induced myocardial ischemia (MSI) for whole body imaging in conjunction with mental arithmetic and control tasks. Mental stress testing was performed by trained staff using mental arithmetic. First, participants were asked to count out loud for the mental arithmetic control condition. For the mental arithmetic stress condition they were asked to perform a series of increasingly complicated mathematical calculations under time pressure, including addition, subtraction, multiplication and division, while they received negative feedback on their performance from a staff member performing the test who was wearing a white coat ^100^ PET imaging of the brain was performed with a Siemens whole body PET camera (Siemens, Inc, Erlangen, Germany). Blood pressure and heart rate were recorded at 5-minute intervals during the resting phase and at 1-minute intervals during the stress phases using an automatic oscillometric device.

Participants underwent two PET scans of the brain, heart, and whole body, in conjunction with control and stressful tasks. During each scan, radiolabeled glucose ([^8^F]2-fluoro-2-deoxyglucose (FDG)), produced in an on-site cyclotron, was injected for measurement of glucose uptake. During the first scan, patients were asked to count out loud. The second scan was performed during an acute mental stress challenge involving mental arithmetic. All sessions lasted for 5 minutes and 20mCi of FDG was injected 10 seconds after each task started.

### Image Analysis

PET images of the brain, heart and whole body were reconstructed. Images were normalized using patient body weight and glucose levels in the blood. Regions of interest in the brain were determined using the Automatic Anatomic Labelling masks.^102^ Regions of interest were placed on the heart using standard 17-segment templates. Regions were also placed over the aorta, liver, spleen, adipose tissue and bone marrow. A region was placed on the left gluteal subcutaneous adipose tissue and over the abdominal aorta anterior to the L4 vertebral body. Analysis of PET brain images, including realigning, normalizing, and smoothing, was completed following established protocols^103-105^ using statistical parametric mapping (SPM12). Automatic Anatomic Labeling atlas was applied to the rest and stress images.^106,107^

### Statistical Analysis

Analysis of variance (ANOVA) was used to compare baseline demographic and risk factors. The relationship between brain regional glucose uptake and glucose uptake in peripheral organs for mental stress and neutral conditions was examined using linear regression.

## Results

Demographic and risk factors, including age, sex, race, depressive symptoms, body mass index (BMI), history of smoking, diabetes, hypertension, or dyslipidemia, as well as patterns for use of medications including vasodilators, angiotensin receptor blocker, angiotensin converting enzyme inhibitors, diuretics and beta-blockers, are presented in Table 1.

**Figure 2.**
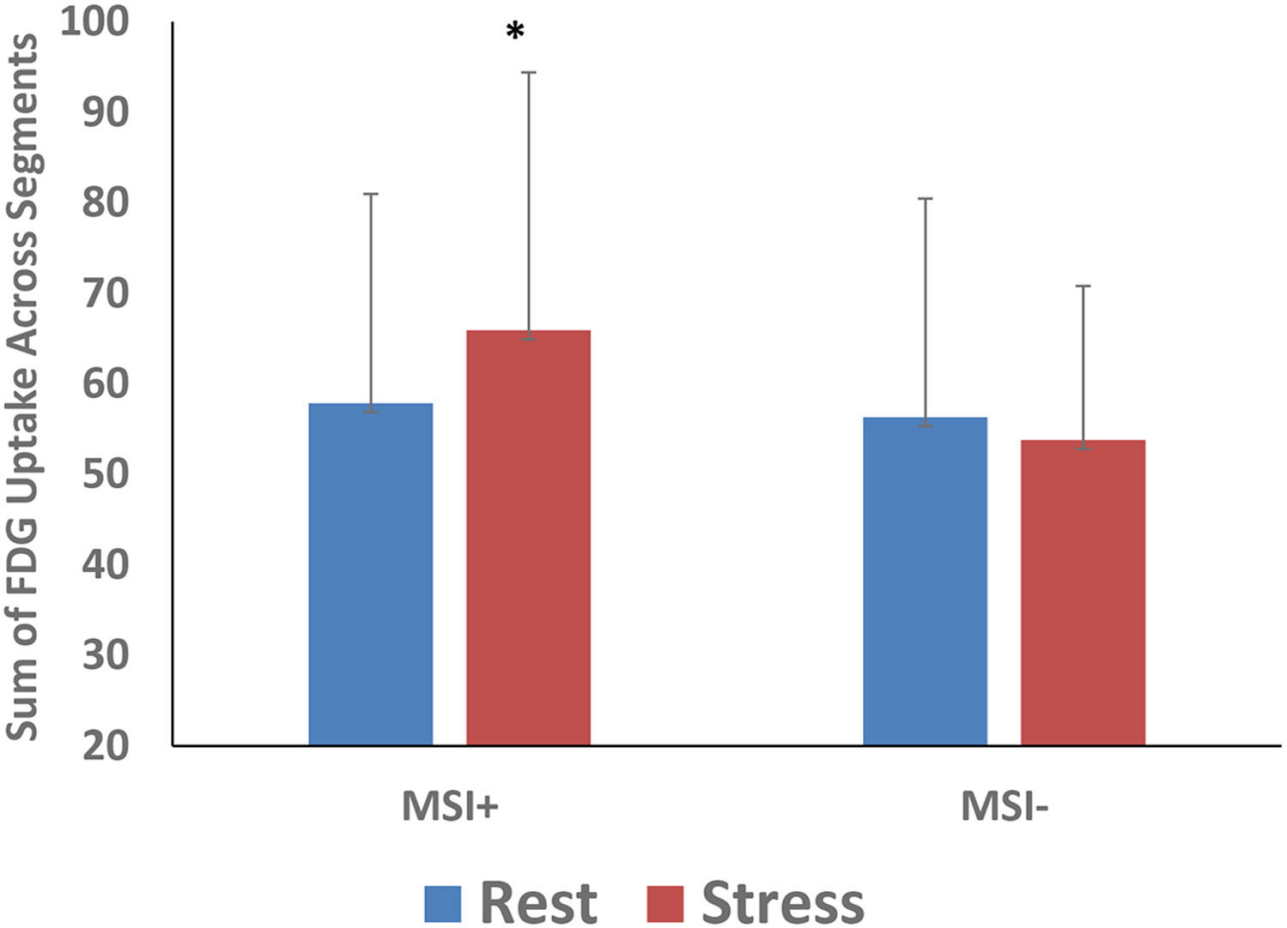
Effect of mental stress on cardiac glucose uptake. There was an 18% greater increase in glucose uptake with stress versus rest in the MSI+ (N=4) compared to MSI− CHD (N=6) patients (*p=.19).

Patients with MSI compared to those without MSI showed a pattern of increased uptake in the heart (Figure 2) (18% greater), medial prefrontal cortex (7%), and adipose tissue (38%) with stress. In the CHD group as a whole, there was a 5% increase in spleen and 21% increase in the mediastinum with stress. Glucose metabolism at rest and with mental stress in brain areas mediating stress and emotion and hypothesized to mediate the effects of stress on CHD was correlated with glucose uptake in peripheral organs involved in inflammation and regenerative capacity, including spleen, liver, bone marrow, and mediastinum. Glucose uptake in these brain areas also showed correlations with uptake in the adipose tissue, known to have high pro-inflammatory activity, and with aorta, suggesting vascular inflammation (Table 2). For instance, resting metabolism in several medial prefrontal/anterior cingulate was correlated with glucose uptake at rest in bone marrow, mediastinum, liver, aorta and spleen, while amygdala metabolism was correlated with resting bone marrow uptake, insula was correlated with adipose tissue, and thalamus correlated with liver, aorta and spleen (Table 2a). Resting glucose metabolism in medial prefrontal/anterior cingulate areas was also correlated with post-stress glucose uptake in bone marrow, mediastinum, liver, aorta, adipose tissue, and spleen (Table 2b).

**Figure 3.**
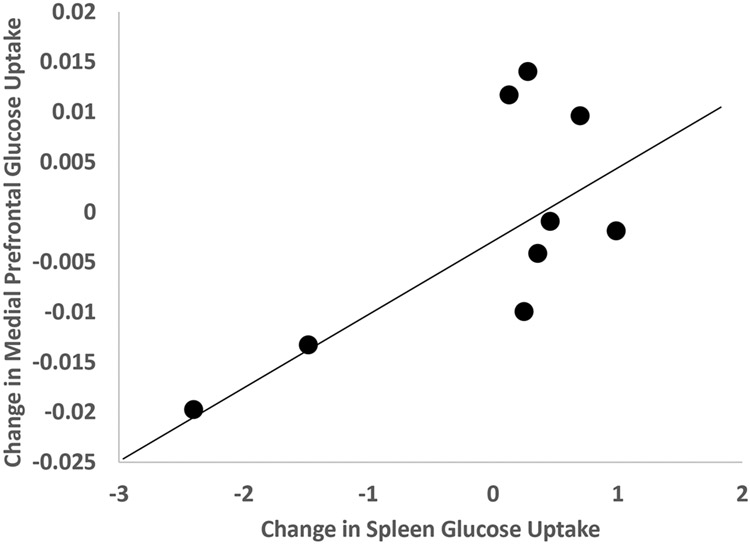
Relationship between change in glucose in the medial prefrontal cortex with mental stress and change in spleen glucose uptake with mental stress in CHD patients (r=0.69, p0.038).

**Figure 4.**
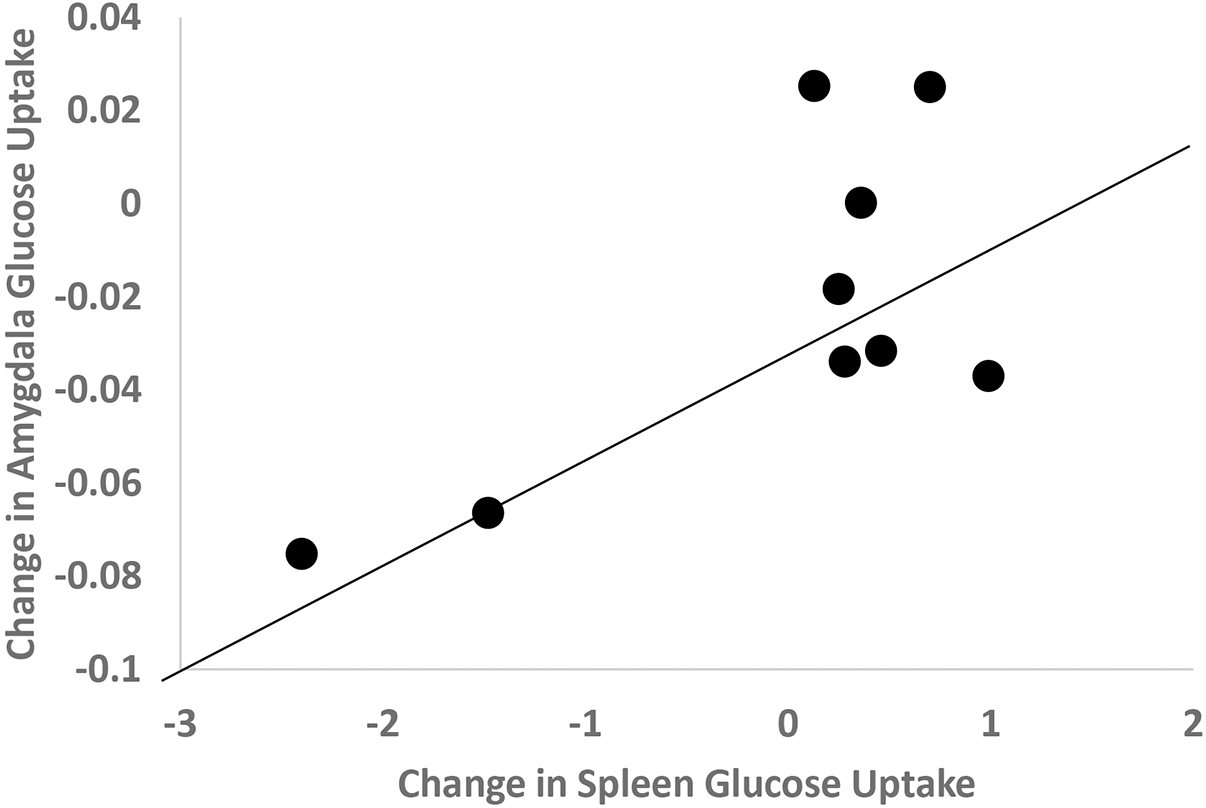
Relationship between change in amygdala glucose uptake with mental stress and change in spleen glucose uptake with mental stress (r=.69, p=.04).

**Figure 5.**
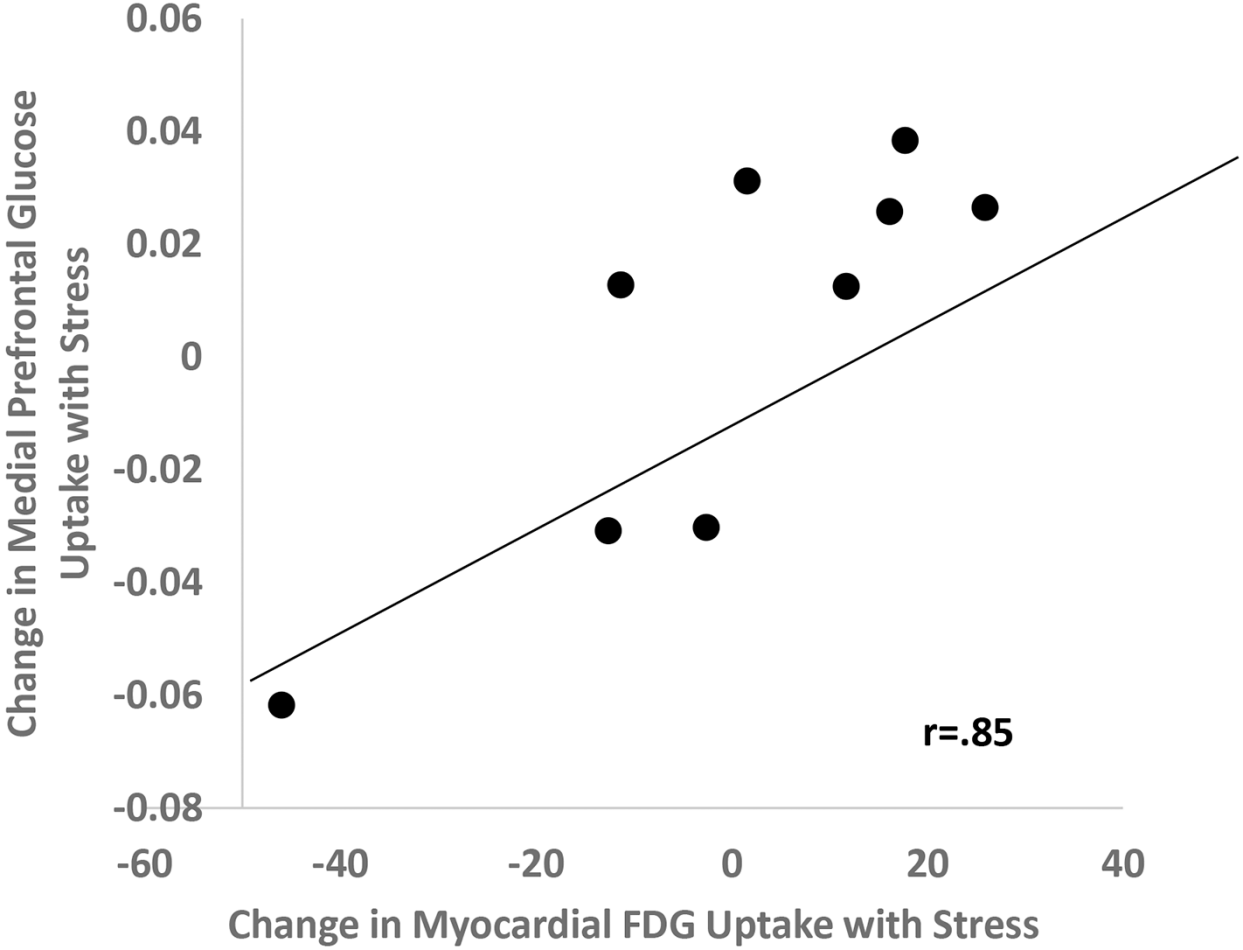
Relationship between change in medial prefrontal cortex (superior orbital) glucose uptake and change in myocardial uptake with mental stress (r=.85, p=.004).

Post-stress glucose metabolism in brain areas involved in visual perception (inferior parietal lobule, cuneus, occipital cortex) correlated with post-stress bone marrow uptake, and in the case of cuneus also with mediastinum uptake (Table 2c). The increase (delta) in glucose metabolism with stress in medial prefrontal/anterior cingulate areas correlated with the increase (delta) in glucose uptake with stress in liver, aorta (Table 2d), and spleen (Figure 3, Table 2d) Other correlations for the delta change with stress included olfactory cortex with bone marrow, visual processing areas (lingual gyrus, calcarine) with adipose tissue, parahippocampal gyrus with spleen, temporal pole with bone marrow and aorta (Table 2d), and amygdala with spleen (Figure 4, Table 2d). The change in medial prefrontal/anterior cingulate glucose metabolism with stress also correlated with the change in glucose in the myocardium with stress (Figure 5, Table 2d). Other brain areas not implicated in stress (e.g. caudate, putamen) did not show similar correlations.

## Discussion

This study showed a relationship in CHD patients between glucose uptake in brain areas that mediate mood, emotion and the stress response, including medial prefrontal cortex, insula, and amygdala, and uptake in peripheral tissues that indicate increased inflammation (aorta) and regenerative activity/inflammation (bone marrow, mediastinum, liver, spleen) following exposure to mental stress. There was also a relationship between mental stress-induced increased activity in medial prefrontal cortex and mental stress-induced increases in cardiac glucose uptake, indicative of myocardial ischemia.

This study adds to the literature that shows a relationship between function of brain areas involved in mood, stress and emotion, peripheral organ systems involved in inflammation, immune function, regenerative capacity, adiposity and the myocardium, in CHD patients.^35,36,42,88^ The current study extends the literature by being the first to look at an acute stress paradigm (mental stress) in the laboratory. This adds to our previous findings that medial prefrontal cortex function with stress is linked to adverse outcomes in CHD, an effect mediated by reduced heart rate variability (HRV) and increased inflammation (interleukin-6 (IL-6))^102^ as well as other studies in the literature that found an association between increased amygdala activity and subjective stress and adverse cardiac outcomes in CHD patients.^91,93,108^ Similar to our study, in that prior study, brain activity was correlated with activity in adipose tissue (although visceral, not subcutaneous) and bone marrow, as well as inflammation.^88,109^ Together findings from the two studies, ours with rest and acute stress and the prior one at rest, suggest that activity in brain areas mediating emotion and fear in stress-vulnerable CHD patients drive inflammation, which in turn results in increased regenerative capacity and intraabdominal adiposity.^88,109^ These findings highlight mechanisms involving brain, inflammatory, and autonomic responses by which stress increases risk for CHD, suggesting targets for treatment intervention.

These findings suggest important mechanisms through which stress acts through the brain and potentially mediates CHD risk through peripheral organs involved in inflammatory and regenerative processes. The inflammatory system, which includes pro-inflammatory biomarkers like interleukin-6 (IL-6) and interferon-γ (IFN-γ), in addition to fighting infections is also responsive to stress. Increases in inflammation are associated with stress-related psychiatric disorders,^110-116^ and catecholamines, released during stress, represent a key link between stress and CHD ^117^ Catecholamines released as part of the fight-or-flight response, in both animal studies and with mental stress tasks in the laboratory, act through adrenergic receptors to activate the transcription factor, nuclear factor-κB (NF-κB), which leads to increases in inflammatory cytokines,^118^ This is largely mediated through sympathetic neurons that terminate in the spleen, acting through cholinergic neurons to activate cell mediated immunity.^119^

The spleen plays a key role in cell mediated immunity.^120,121^ Arterioles from the splenic artery branch in the trabeculae of the spleen and arrive in the White Pulp (WP), which contains B cells, T cells, and Dendritic Cells (DCs), and mediates production of antibodies.^119^ Blood then flows to the Red Pulp (RP) area which mediates blood filtration, phagocytosis of old erythrocytes, iron recycling and response to bacterial infiltration.^119^ In the Marginal Zone (MZ), which lies between the WP and the RP, B cells are activated to produce immunoglobulins (IgA, IgM, and IgG). Cell mediated immunity utilizes T cells including CD8 cytotoxic cells and CD4+ cells that both produce cytokines and engulf microbes. While catecholaminergic fibers terminating in the spleen activate cytokine production, the vagus nerve acts through cholinergic neurotransmission to have an anti-inflammatory effect through the spleen, including inhibition of production of TNF-α by macrophages in the RP and MZ^122-126^ and regulation of B and T lymphocytes in the WP.^127^ The current study found medial prefrontal cortex brain activity with rest was correlated with spleen glucose uptake both at rest and following mental stress tasks, and increases in this brain area with stress correlated with increases with stress in spleen. We have previously shown increased activity in this area in CHD patients with mental stress-induced ischemia^100^ and shown a correlation with future adverse CHD outcomes mediated in part by increased inflammation (IL-6).^102^ Frontal cortex changes with stress correlated with changes in spleen. Mental stress induced amygdala activation correlated with stress-induced spleen activity. Both medial prefrontal cortex and amygdala have direct and indirect (through the hypothalamus) connections to peripheral sympathetic pathways implicated in mechanisms of stress on CHD risk. The current findings suggest these brain areas may mediate activation of the spleen and hence inflammation through sympathetic autonomic pathways.

Other peripheral organs play a role in inflammatory responses and/or regenerative activity. Both liver and bone marrow are a source of lymphocytes.^121^ Bone marrow is also a source of regeneration of cells. We have used telomere length and/or circulating progenitor cells (CPCs) as markers of stress, accelerated aging and/or regenerative capacity, and showed they predicted future adverse cardiovascular events,^128^ myocardial ischemia,^129^ worse cognitive function,^130^ and adverse cardiovascular outcomes in patients with CHD.^131^ The current findings suggest that stress-induced activation in medial prefrontal cortex/anterior cingulate activates inflammatory and regenerative activity in peripheral organs.

This study found a relationship between activity in brain areas hypothesized to underlie CHD risk and glucose uptake in adipose tissue. Obesity is a known risk factor in CHD, but it has also been shown to be associated with alterations in inflammation, possibly mediated via the liver and spleen.^121^ The current study showed increased glucose uptake with stress in adipose tissue, especially in CHD patients with MSI, while other studies showed increased amygdala activity and subjective stress correlated with both visceral fat and inflammation as well as adverse cardiac outcomes.^91,93,108^ It is possible that brain areas mediating emotion and fear stimulate inflammation which in turn results in increased intraabdominal adiposity.^88,109^ Those studies showed visceral adipose tissue volume was correlated with both arterial inflammation measured by FDG uptake and adverse cardiac events in patients with CHD.^109^

The current study has several important limitations. The sample size was small, and the ability to compare CHD patients with and without mental stress-induced myocardial ischemia was even more limited. This was a pilot study whose purpose was to make an initial examination of whether brain activity with stress was associated with changes in function in peripheral organs involved in inflammation and regenerative activity in a population of Coronary Heart Disease patients felt to be most at risk of having a pathway of stress acting through brain areas involved in emotion and stress to activate peripheral inflammation and regenerative functions that could in turn have effects on the heart. A limitation of the study is the lack of normal control subjects without CHD, therefore it cannot be concluded that the findings of the current study are specific to CHD patients. Future studies should include such controls. Strengths of this pilot study include imaging of brain, heart, and multiple peripheral organs involved in inflammation and regenerative capacity simultaneously, at rest and with an acute stress delivered in the laboratory. This work should be replicated in a larger sample.

## Conclusion

Brain activation in brain areas mediating stress and emotion was associated with increased activity in peripheral organs mediating inflammation and regenerative activity in patients with Coronary Heart Disease. These findings suggest that stress acts through the brain to activate peripheral inflammatory and regenerative pathways, providing a mechanism for how stress may increase heart disease risk. These findings suggest future targets for intervention to reduce the risks of stress and related conditions like depression and posttraumatic stress disorder for increasing morbidity and mortality related to Coronary Heart Disease.

## Figures and Tables

**Table 1.** Demographic and Risk Factors for CHD Patients

Demographics	N	N = 10^1^

**Age**	10	56 (53, 58)
**Female**	10	50% [5 / 10]
**Race**	10	
Black		50% [5 / 10]
White		50% [5 / 10]
**BMI**	10	30.3 (24.6, 37.3)
**Health History**		
**Beck Depression Inventory**	10	6 (2, 10)
**Hypertension, ever**	10	50% [5 / 10]
**Dyslipidemia, ever**	10	60% [6 / 10]
**Diabetes, ever**	10	20% [2 / 10]
**Medications**		
Antidepressants	10	30% [3 / 10]
ACE inhibitors	10	40% [4 / 10]
Anxiolytics	10	10% [1 / 10]
AR blockers	10	20% [2 / 10]
Beta blockers	10	80% [8 / 10]
Diuretics	10	40% [4 / 10]
Vasodilators	10	10% [1 / 10]
**Clinical outcomes**		
**Rate-Pressure Product, speech task**	10	11,817 (10,885, 13,714)
**Rate-Pressure Product, math task**	10	11,382 (10,650, 12,580)
**PAT ratio, speech task^2^**	8	0.58 (0.54, 0.72)

**Table 2.** Relationship between Resting and Post-Stress Brain Metabolism and Peripheral Organ Glucose Uptake

a. Relationship between Resting Brain Metabolism and Resting Peripheral Organ Glucose Uptake							
	Bone Marrow	Mediastinum	Liver	Aorta	Adipose Tissue	Spleen	Myocardium
Ant. Cingulum	0.71 (p=0.03)						
Frontal Med. Orbital	0.76 (p=0.01)	0.67 (p=0.046)	0.91 (p=0.0006)	0.83 (p=0.005)		0.88 (p=0.001)	
Insula					0.78 (p=0.01)		
Thalamus			0.78 (p=0.01)	0.69 (p=0.04)		0.83 (p=0.006)	
Amygdala	0.68 (p=0.04)			0.65 (p=0.56)		0.65 (p=0.57)	
b. Relationship between Resting Brain Metabolism and Post-Stress Peripheral Organ Glucose Uptake							
	Bone Marrow	Mediastinum	Liver	Aorta	Adipose Tissue	Spleen	Myocardium
Ant. Cingulum				0.68 (p=0.04)			
Frontal Inf. Orbital			0.86 (P=0.002)		0.79 (p=0.01)	0.82 (p=0.006)	
Frontal Med. Orb.	0.76 (p=0.01)			0.86 (p=0.002)			
Frontal Sup. Orb.		0.7 (p=0.037)					
Gyrus Rectus	0.72 (p=0.28)				0.72 (p=0.028)		
c. Relationship between Post-Stress Brain Metabolism and Post-Stress Peripheral Organ Glucose Uptake							
	Bone Marrow	Mediastinum					
Inf. Parietal	0.7 (p=0.02)						
Cuneus	0.8 (p=0.005)	0.64 (p=0.047					
Occipital	0.8 (p=0.005)						
d. Relationship between Change (Increase) in Brain Metabolism and Change (Increase) in Peripheral Organ Glucose Uptake With Stress							
	Bone Marrow	Mediastinum	Liver	Aorta	Adipose Tissue	Spleen	Myocardium
Ant. Cingulum							0.67 (p=0.04)
Frontal Inferior			0.77 (p=0.038)				
Frontal Lobe							0.84 (p=0.004)
Olfactory Ctx	0.78 (p=0.01)						
Lingual				0.71 (p=0.03)			
Parahippocampal Gyrus						0.75 (p=0.018)	
Temporal Pole	0.81 (p=0.007)			0.91 (p=0.0005)			
Amygdala						0.69 (p=0.04)	
Calcarine		0.75 (p=0.02)			0.73 (p=0.02)		

Ant.=Anterior; Ctx.=Cortex; Inf.=Inferior; Orb.=Orbital; Sup.=Superior

## Abbreviations:

AA: African American
ACE: angiotensin converting enzyme
ACS: Acute Coronary Syndrome
BDI: Beck Depression Inventory
BMI: body mass index
CAD: coronary artery disease
DSM: Diagnostic and Statistical Manual
F: female
FDG: [F-188 2-fluoro-2-deoxyglucose
HRPET: High Resolution PET
HRRT: High Resolution Research Tomograph
IRB: Investigational Review Board
M: male
MI: myocardial infarction
MIPS: Mental Stress Ischemia and Prognosis Study
MSI: Mental Stress Ischemia
NA: Native American
PAT: Peripheral Arterial Tonometry
PET: Positron Emission Tomography
PTSD: posttraumatic stress disorder
SCID: Structured Clinical Interview for DSM
SPECT: Single Photon Emission Tomography
spm: statistical parametric mapping
SUDS: Subjective Units of Distress Scale
VA: Veterans Administration
